# Toward Healthy Aging in Palau

**DOI:** 10.1093/geront/gnad078

**Published:** 2023-06-23

**Authors:** Wenqian Xu, Tarita Holm, Siwon Lee, Gaafar Uherbelau, Sherilynn Madraisau, Hera Subediang

**Affiliations:** World Health Organization Regional Office for the Western Pacific, Manila, Philippines; Department of Health Sciences, Lund University, Lund, Sweden; World Health Organization Regional Office for the Western Pacific, Manila, Philippines; World Health Organization Regional Office for the Western Pacific, Manila, Philippines; Ministry of Health and Human Services, Koror, Palau; Ministry of Health and Human Services, Koror, Palau; Ministry of Health and Human Services, Koror, Palau

**Keywords:** Age-friendly environment, Health system transformation, Multisectoral collaboration, National policy

## Abstract

**Background and Objectives:**

Palau is facing a rapidly aging population and developing a comprehensive national aging policy to address the growing needs of older adults, but more research is needed to understand their circumstances and needs in relation to healthy aging. This study aims to investigate these factors and contribute to developing the National Policy on Care for the Aging.

**Research Design and Methods:**

The study adopted a participatory action research design and included focus groups with older adults, public consultations, and stakeholder interviews, providing insights into the needs of an aging population and how to address them. Thematic analysis was conducted to understand the experiences and needs of older adults and possible system changes to address these needs.

**Results:**

This study identified the need to recognize cultural changes and optimize social and physical environments to improve the health and well-being of older adults. Older adults are valued in Palauan society, but harmful stereotypes and mistreatment of older adults exist. To address those needs and challenges, our stakeholders proposed a range of services, programs, and policies across sectors to create a healthy-enabling environment. Our findings also highlight the importance of health systems proactively reaching individuals and families to address lifelong health needs.

**Discussion and Implications:**

Our results show the vital role of various sectors in fostering healthy aging and the importance of environmental and cultural perspectives in creating an age-friendly society, which can be an inspiration for other Pacific Island countries.

## Background and Objectives

The Republic of Palau ranks 80th in the United Nations Development Programme (2022) and has a population of 17,614 people as per the 2020 census. As one of the highest income-generating Pacific Island states, Palau boasts a per capita gross domestic product of $12,083 (2021). Palauan and English are its official languages, with Palauan being the largest ethnic group at 70.6% and Asians being the second largest at 26.5% (Republic of Palau Office of Planning and Statistics, 2022). Eighty-one percent of the population lived in urban areas in 2021 (United Nations Population Division, 2018).

Palau is experiencing a rapidly aging population, with the percentage of people aged 60 and older projected to increase from 9.9% to 24.6% by 2050 (United Nations Department of Economic and Social Affairs Population Division, 2022). Palau is aging faster than most Pacific Island countries and territories and is expected to become an aged society by 2033, with those aged 65 and older accounting for over 14% of the population. The country is also projected to have a high proportion of the oldest old (United Nations Population Fund Pacific Sub-Regional Office, 2014).

Population aging poses significant challenges for both individuals and Palauan society. The increasing burden of noncommunicable diseases (NCDs) is a major public health concern in the country, accounting for 65% of hospital costs and almost 90% of medical referral costs (Institute for Health Metrics and Evaluation, 2019b). Further, population aging has led to changing and diverse demands for goods and services, such as health, long-term care, social protection, and information and communication. It may also shape family structures and intergenerational ties and affect the way in which individuals and communities plan for aging. In response, in 2020, the President of Palau signed a bill to develop a comprehensive National Policy on Care for the Aging to address the growing and diverse needs of older adults.

Healthy aging can turn challenges associated with population aging into the opportunities that come with this stage of life, such as increased experience and contributions to families and society (World Health Organization Regional Office for the Western Pacific, 2020). Healthy aging refers to “the process of developing and maintaining the functional ability that enables well-being in older age” (World Health Organization, 2021); its focus is on optimizing environments across life courses toward well-being (Keating, 2022). Healthy aging is affected by sociodemographic, biological, behavioral and psychological, and social determinants (Kralj et al., 2017). As synthesized by a literature review, the social factors affecting older persons’ health and well-being include physical activity, diet, self-awareness, outlook/attitude, lifelong learning, faith, social support, financial security, community engagement, and independence (Abud et al., 2022). Given that various environmental factors that affect the health of older populations exist beyond the health sector, it is crucial to not only transform health systems to enhance the functional abilities of older adults but also to create health-enabling environments that support healthy aging (Xu et al., 2023). In light of this, the present study adopts the public health framework for healthy aging (World Health Organization, 2021), which presents opportunities for public health actions across the life course, to research the current status of and potential changes in belief, social, and health systems that support healthy aging in Palau.

Given the lack of studies exploring the situation of older adults in Palau and the Pacific Islands region in general, this study aims to investigate the circumstances, needs, and preferences of older adults in relation to various aspects of healthy aging in the country. The results of this study will contribute to a better understanding of the needs and circumstances of older adults and support the development of the first national aging policy. The outcomes of this research may inform policy strategies and options suited to the national and subnational contexts in Palau. The study aims to answer two research questions:

1. What are the preferences, needs, and expectations of older adults regarding health and well-being, from their perspective?
2. How can policymakers, communities, families, and young generations respond to the identified needs of older adults?

## Research Design and Methods

This study adopts a participatory action research (PAR) design, which entails a systematic inquiry that involves the participation of those affected by the issue being studied. The goal of this approach is to promote education and action that leads to social change (Green et al., 1995). The PAR design is characterized by its core principles, such as coresearcher engagement, an empowering and colearning process, capacity building, and systems change (Blair & Minkler, 2009). In line with the PAR approach, we ensured the meaningful participation of older adults and relevant stakeholders in several aspects of the study. These include data collection and interpretation, dissemination of research findings at different phases, and using the findings as a basis for developing the National Policy on Care for the Aging and discussing priority activities and programs for implementation in the short term. Research findings were used to draft the National Policy on Care for the Aging in collaboration with interested study participants and policymakers, which will help transform systems that better support healthy aging. Throughout the policy development process, study participants provided support by reviewing draft versions, offering additional information, and suggesting revisions at several meetings with the Coordinating Committee on Care for the Aging (CCCA) and through e-mail communications. In addition, study participants also contributed to increasing public knowledge of societal aging and planning for the future implementation of this policy. The President of Palau signed this newly developed policy on April 27, 2023, and it will be presented to the National Congress. The findings of this study and the policy are immediately contributing to designing pilot aging programs.

This study was divided into two components, each aimed at addressing specific research questions.

### Focus Group With Older Adults

The first component involves exploring the preferences, needs, and expectations of older adults with regard to health and well-being, as well as their desired contributions to families, communities, and society. To achieve this, a qualitative descriptive method, which involves conducting focus-group discussions, was selected. The focus groups are guided by a moderator and observed by research assistants. This method allows for in-depth knowledge to be generated from older adults by gathering and understanding their thoughts and experiences. Consecutive sampling was used to recruit adults aged 55 years and older, who are living in their own homes and come from 16 states across Palau. Participants were approached through the Aging Council, consisting of representatives from each of the country’s states. The aim was to recruit a diverse group of older adults, including different backgrounds in terms of age, gender, marital status, language, and household composition. Only those who gave informed consent and met the inclusion criteria were recruited and given a written information sheet. The focus groups were conducted between October and December 2022 in a hotel conference room involving 37 older adult participants (see Table 1), using both English and Palauan languages. A preliminary pilot focus group was conducted with three older adults, but its data were not included in the analysis. In total, six focus groups were conducted, with four groups consisting of Palauan older adults and two groups consisting of non-Palauan older adults. The discussions were audio recorded and lasted approximately 2 hr and were guided by an interview guide (see Supplementary Material 1). Field notes taken during the focus groups were analyzed to evaluate the theoretical saturation of the data by two authors. Saturation was reached when no new relevant information related to research questions was produced by the participants. Data were transcribed verbatim and translated into English as needed for data analysis.

**Table 1.** Descriptive Characteristics of Focus Group Participants

Demographic characteristics		Frequency (*n* = 37)	Percentage (%)
Age	55–59	5	13.5
	60–69	24	64.9
	70–79	6	16.2
	80 and older	1	2.7
	Unspecified	1	2.7
Gender	Female	18	48.6
	Male	19	51.4
Ethnicity	Palauan	25	67.6
	Non-Palauan	12	32.4
Marital status	Single	4	10.8
	Married	26	70.3
	Divorced	3	8.1
	Widowed	4	10.8
Education	Elementary	1	2.7
	High school	4	10.8
	Some college	11	29.7
	College degree	21	56.8

### Stakeholder Consultations and Interviews

The second component of the study aimed to gather perspectives from various stakeholders through stakeholder consultations and interviews (see Table 2) on how to address the needs and preferences of older adults. Stakeholder consultations were held during the national symposium on healthy aging on December 16, 2022, with over 70 registered participants from all states of Palau, including policymakers, nonprofit organizations, health care and social workers, community leaders, and youth representatives. The symposium included two group discussion sessions: one that explored the current situation of older adults and another that envisioned an aging society in six thematic areas. The six areas were identified based on the data collected from focus group participants using thematic analysis (for more information on how they were identified, please refer to the *Data Analysis* section). Instructions for the group discussions were provided (refer to Supplementary Material 2 and 3). The approved summary report of this symposium was produced for data analysis. For the stakeholder interviews, purposive sampling was utilized in collaboration with the Ministry of Health and Human Services to identify and recruit relevant stakeholders. Information about the study and its purpose was provided and voluntary participation was solicited. Ten in-depth semistructured interviews were conducted with stakeholders from the active and healthy aging arena, such as family caregivers, community leaders, health care and social care providers, businesses, and policymakers, from December 14 to 20, 2022 (for the interview guide, see Supplementary Material 4). These interviews provided valuable perspectives and insights on how to address the diverse needs of an aging population. The interviews lasted between 30 to 60 min, and field notes were taken during the interviews for data analysis. The stakeholders were informed by the preliminary results of focus-group interviews with older adults (see Supplementary Material 5).

**Table 2.** Descriptive Characteristics of Stakeholders

Public consultations			In-depth interview participants			
Type of participants	Number of participants	Gender distribution	ID	Sector	Gender	Main areas of business
National government entity	12	9 male; 3 female	1	Private sector	Male	Local media and an interest in long-term care facilities
Ministry of Health and Human Services	18	5 male; 13 female	2	Community	Female	Social programs and services for older adults
National congress	3	3 male	3	Government	Male	Legislative affairs
Private sector	4	3 male; 1 female	4	Government	Female	Public health policy
Civil society	13	4 male; 9 female	5	Health care	Female	Rehabilitation and nursing
Youth representatives	4	1 male; 3 female	6	Health care	Female	Geriatrics and community health
Other (not known)	5	4 male; 1 female	7	Government	Female	Cultural and community affairs
			8	Family	Female	Family caregiving
			9	Nonprofit sector	Female	Aging programs for rural communities
			10	Public authority	Female	Community services and activities

### Data Analysis

The data analysis consisted of two components. First, a thematic analysis (developed by Braun & Clarke, 2006) was conducted on data from focus group interviews with older adults to address research question 1, which explored the preferences, needs, and expectations of older adults regarding their health and well-being. A four-step analytical process, similar to that employed by Salma and Salami (2020), was followed to interpret the data, involving gaining a comprehensive understanding of the experiences and needs of older adults, synthesizing the data to uncover patterns, using the Regional Action Plan on Healthy Aging (World Health Organization Regional Office for the Western Pacific, 2020) to explain the identified patterns and organize them into six thematic areas (related to social and health systems), and engaging in a dialogue with CCCA and other relevant stakeholders to assess and verify the relevance and meaning of the preliminary results (as shown in Supplementary Material 5). The process of verifying preliminary findings involves identifying any inconsistencies or disparities between the findings from focus group participants and the dialogs with stakeholders, analyzing the underlying reasons for these differences in experiences and perspectives, assessing the implications of such discrepancies for the development of a national aging policy, and revising the preliminary findings based on the feedback provided by stakeholders. Stakeholders did not raise objections to the revised findings related to the needs and preferences of older adults. The validation of the findings regarding the needs of older adults was performed before the stakeholder consultations.

Second, a thematic analysis was conducted on data collected from stakeholder consultations and interviews, with a focus on analyzing how society can address the needs of older adults identified in the first component of data analysis. These findings were organized thematically in accordance with the findings of the first part of the analysis and discussed and verified with policymakers. ATLAS.ti qualitative software version 8.4.4 was used for data analyses.

## Results

We identified four thematic areas where the needs and expectations of older adults become evident, along with the potential interventions to address them from the perspective of stakeholders (including older adults).

### Recognizing Cultural Changes Around Aging and Older Adults

Palauan society has a long-standing tradition of valuing and respecting older adults. They play a crucial role in their families, communities, and society, serving as caregivers and leaders while passing on their knowledge, experiences, and skills to younger generations. Unfortunately, instances of abuse of older adults and ageist perceptions and behaviors are on the rise. Some stakeholders expressed that mistreatment, including neglect and abuse, of older adults in long-term care settings occurred but was rarely reported (Reklai, 2021). These instances are having negative effects on both families and communities. The culture of respecting older adults is declining, with harmful stereotypes labeling older people as frail, dependent, and incapable becoming more prevalent. As indicated in the following comment by Participant 27, this sometimes leads to compassionate ageism, which prevents older adults from participating in activities that are beneficial to their health and well-being, causing them to feel like a burden.

I think young adults are still respectful; it’s when you get down to the little kids that [inaudible], it’s hard to get respect from them, but, you know, young adults still. What I think is people thinking you can’t do things, can I help you with this or with this, no I am quite capable of doing things. (Participant 27)

To address these issues, it is necessary to thoroughly analyze and understand the contributing factors. According to stakeholders, potential drivers of these cultural changes include external cultural influences (such as socialization from Western media, new economic conditions and disparities, and outmigration of Palauans from their communities). Additionally, Palauan society is expected to maintain its tradition of respecting older individuals and fostering positive intergenerational relationships. Stakeholders believe that Palauan society should improve public understanding of older adults and tackle internalized age stereotypes to empower older adults to take control of their lives and build confidence. It is important for present generations and local communities to practice and improve the quality of respect for older adults, instilling these values in the next generation. As expressed by stakeholders, intergenerational activities are needed to strengthen social connections and support the transmission of knowledge, values, culture, and experiences.

To tackle ageism, the possible actions include encouraging the older generation to recognize themselves as leaders and advisors in community work, involving the community in caring for the needs of older generations, making it “everybody’s business/role,” creating intergenerational programs, preserving and maintaining existing cultural values, and promoting and implementing Palauan values in the home, school and social environment. (Stakeholder consultation with groups 3 and 4)

### Creating Age-Friendly Social Environments

The social factors of employment, civic participation, social participation, and access to technology have a significant impact on the health and well-being of older adults in Palau. Older adult participants expressed that their social needs and expectations are not adequately met. In terms of employment and civic participation, some older adults expressed the need for support systems within the labor market to accommodate their desire to pursue new career paths or continue working in a flexible capacity after retirement. Although many older adults are actively involved in civic activities, such as volunteering and community engagement, they often go uncompensated for their contributions. Furthermore, the following excerpt illustrates that some older adults voiced concerns about loneliness and social isolation in later life and emphasized the importance of social participation opportunities, such as cultural, sports, and health promotion activities. Digital inclusion has also become increasingly important for older adults in contemporary Palau, as technology plays a significant role in interactions with health and social systems. However, despite technological advancements, many older Palauans face digital barriers that limit their civic engagement and access to information and services.

I want to go to the garden/farm to clear and plow and plant tapioca. But I also get bored, because I am alone listening to the animal sounds and the heat is too much. I think to myself that maybe I need to start involving myself in those activities, different activities. (Participant 11)

Over the past three decades, life expectancy at birth in Palau has increased by 2.6 years, including 1.7 years of healthy living and 0.9 years of less healthy periods (Institute for Health Metrics and Evaluation, 2019a). This presents new opportunities for individuals to lead a fulfilling later life. Community-based aging programs played a crucial role in supporting older people to fully participate in society. The Ministry of Community and Cultural Affairs provided meals for homebound older adults and Old Age Center attendees, assistive devices for low-income older adults through home visits, and transportation services; additionally, they organized social and intergenerational activities such as traditional cultural activities, cultural exchange with foreign visitors, physical exercise programs, and national holiday events for older adults, with partner agencies (Ministry of Community & Cultural Affairs, 2020). Nevertheless, ensuring the continuity of these programs remains a challenge due to funding interruptions.

To address the needs and expectations of an aging population for age-friendly social environments, stakeholders believe it is crucial to create an environment that empowers individuals of all ages to take control of their lives. The following excerpt illustrates that this can be achieved by defining and encouraging meaningful roles for older adults in the economy and society, according to both personal preferences and societal expectations. To support this, stakeholders suggest implementing a range of supportive interventions, such as providing adult education, seminars, and training, as well as informal learning opportunities that foster knowledge-based skills. Financial support can be offered to offset costs incurred by older adults participating in volunteer activities. Additionally, digital literacy training programs can help overcome some of the barriers to digital information, services, and social participation that older adults face.

To optimize social environments for older people, a possible action is to partner with education to create work opportunities such as after-school programs and inter-session break programs that involve both students and older adults. These programs may include arts and crafts, tutoring and storytelling, to leverage the potential of an aging workforce. (Stakeholder consultation with groups 1 and 2)

### Developing Health-Enhancing Physical Environments

The physical environment in Palau is not conducive to positive health and well-being outcomes in later life, according to study participants. Specifically, they identified unmet needs and aspirations in housing, transportation, and outdoor spaces. Housing in Palau is often not designed with accessibility in mind for the oldest members of society or those with mobility issues. This limits their ability to live independently and hinders their well-being. Several pilot projects are currently underway to promote age-friendly homes for older women living in rural areas. While these projects are a positive step toward improving living conditions, a more sustainable approach is needed to be incorporated into the aging policy. In addition, existing neighborhood resources such as parks and public spaces are important for all residents, but they often lack age-friendly features, such as safe and accessible designs. This also pertains to disability policies that aim to promote an inclusive environment, such as the Palau National Disability Policy 2017–2020. Its implementation is crucial, especially in the context of population aging. In terms of public transportation, limited transportation options and associated challenges, particularly in remote communities and outer islands, cause difficulties for older people to access social services and remain socially engaged (as indicated by Participant 12). The public bus system, introduced in May 2022, holds great potential to improve the well-being of older adults by providing age-inclusive services.

For us where we don’t have a pathway because we travel by boat or ship, it’s also hard to set up everything. We need to make sure everyone has transportation. Considering the gas price, water conditions and other alternatives like small aircraft, there are many barriers. (Participant 12)

To address these issues, stakeholders suggest implementing specialized programs to incorporate or maintain age-friendly features. For example, extending home accessibility adaptations to more older adults, such as making doorways wider, and installing grab bars, ramps, stairlifts, and accessible showers. Information about financial assistance for home adaptations should also be available for those facing financial hardship. To address the mobility challenge in later life, accessible, affordable, and efficient public transit systems are necessary to provide a range of transportation options for older adults. Additionally, available sidewalks and walkways, as well as opportunities for physical activity and exposure to nature, were suggested to promote walkability and help address road safety concerns for people of all ages. Implementing such programs may help transform physical infrastructure into a health-enabling environment for healthy aging.

### Transforming Health Systems to Address Individuals’ Lifelong Needs

The data analysis identified four subareas of health systems that are related to healthy aging, including public health, long-term care, health care, and social services. NCDs are a major health concern in Palau and among study participants. Access to preventive health services varies across geographic areas, and older adult participants noted current limitations in coordination, monitoring, and communication of preventive services, as well as the absence of personalized health planning, which led to low health-promoting behaviors. To overcome these limitations, some stakeholders suggested an individualized, goal-based health plan and implementation across the life course. Such health plans may empower individuals to proactively manage their health and prevent chronic conditions by including such as lifestyle adjustments, regular health screenings and assessments, and appropriate vaccinations. As indicated in the following comment by Key Stakeholder 3, the health sector could get closer to the community and further support healthy behavioral changes to ensure positive health outcomes. This preventive approach to healthy aging could help tackle the major health concern of NCDs.

The behavior is by design because we design the environment to reward that behavior. If you go to a shopping center, what percentage of the grocery store is selling vegetables, and how much is canned food? The health sector needs to go to the community. Health is somewhere in the community, in the way we shop, the way we live our lives, the way we go to church and the way we engage in customary traditions. (Key Stakeholder 3)

With regard to long-term care, Palauan families play a crucial role in caring for older individuals. However, there are growing concerns regarding family care for older adults and an expectation of support from the community and society, as indicated by the following stakeholders.

More concern was expressed about those who are bedridden and can’t really do anything—hey can’t participate in these types of activities and often they don’t have kids to help them. These are the ones that should be monitored daily to be helped by the state and community. (Stakeholder consultation with the youth group)

Several initiatives were implemented to support families in meeting the long-term care needs of their loved ones, such as a family caregiver train-the-trainer workshop held in 2011 and a family caregiver training for interested individuals offered by the Palau Community College in collaboration with the Ministry of Health and Human Services (Fernandes et al., 2013). However, the current family care system presents challenges due to the adult population’s occupational responsibilities, as well as the younger generations needing to travel abroad for work or study. These factors resulted in a shortage of available and capable family members or friends to provide care, leading to caregiver burnout and an insufficient provision of informal care. To address these challenges, stakeholder participants proposed alternative care models, such as home care, assisted living, day-care or short-stay services, and long-term care facilities. As indicated in the following comment by stakeholders, mechanisms should be established to ensure quality care, such as the accreditation of professional programs and services, the prevention of abuse and violence of older adults, and others. These suggested actions may help address the growing and diverse long-term care needs in Palau, given the limitations of the current family care system.

Attention to basic human rights and special needs, as well as addressing negative attitudes towards older adults, are crucial. A focal point person is suggested to link all services for improving coordination and collaboration and promoting partnership. (Stakeholder consultation with groups 5 and 6)

Some older adult participants expressed that they experienced early declines in intrinsic capacities, which limited their ability to maintain good health and thrive in society to varying degrees. Given that older adults are prone to multimorbidity and more susceptible to certain diseases and conditions in fast-aging Palau, the current health care system is not equipped to effectively address the complex health needs of current and future older adults. To address these challenges, some stakeholders suggested that the health care system should prioritize early detection, monitoring, and treatment of intrinsic capacity declines by increasing access to basic and appropriate health equipment, medicine, and technology. Palau’s planned introduction of an IT system in 2023 can create accessible and sharable comprehensive health records, which can facilitate person-centered assessment and care. Moreover, a multidisciplinary workforce with the necessary knowledge and skills is needed to support older adults with multimorbidity, geriatric syndromes, declining physical and cognitive function, and poor mental health. Taking these actions could hopefully support the health care system to effectively address the complex and growing health needs of an aging population.

Social support and services are important in promoting good health and well-being, as emphasized by older adult participants. However, not all communities have access to or make use of the services. Although old age centers in Palau can serve as valuable resources, the lack of human and financial resources has resulted in a temporary interruption of aging programs at old age centers. Our stakeholder participants propose that enhancing social services and support is crucial in local communities and society as a whole. Initiatives such as arts and crafts, physical exercise, volunteering and learning opportunities, home-delivered meals, and mental health services can be implemented in the future to promote healthy aging and enhance community development and well-being. These initiatives can possibly strengthen intergenerational connections and foster social support in Palauan communities. In addition, older adult participants suggest other terms to refer to old age centers that may be less stigmatizing and reflect the opportunities for socialization, learning, and wellness activities, such as healthy aging centers or multigenerational community centers.

## Discussion and Implications

This study aimed to investigate the circumstances, needs, and preferences of older adults in relation to various aspects of healthy aging and how to address them in Palau. The most prominent finding to emerge from the analysis is the need for age-friendly Palauan communities involving different environmental aspects that cater to the needs and preferences of older adults. This aligns with the World Health Organization’s concept of an age-friendly environment, which includes social, physical, and service environments that promote health, participation, and security, and enable older adults to age in place (World Health Organization, 2007). The findings highlight the need to address various areas, such as safe and accessible outdoor spaces, transportation options, social participation opportunities, access to health care and support services, and learning and personal development opportunities. As the factors affecting healthy aging span across various sectors beyond just health, addressing them requires a collaborative effort from multiple sectors. In Palau, NCDs account for 70% of deaths and lowering life expectancy (Republic of Palau, 2015). The shift from subsistence living to a more modernized lifestyle and the preservation of certain traditional customs unfavorable to good health has contributed to higher burdens of NCDs and poor mental health in Palau (World Health Organization, 2017a). Because the rise of NCDs in Palau can be attributed to metabolic and behavioral risk factors such as dietary risks and tobacco use (Institute for Health Metrics and Evaluation, 2019a), it is essential that health systems shift their focus from solely treating diseases to promoting health and creating health-enabling environments through prevention. Research highlights that multisectoral action plays a crucial role in creating social, economic, and physical conditions that empower people of all ages to exercise control over their health by adopting healthy lifestyles (World Health Organization, 2021). To achieve this goal, the health sector must partner with the community and other relevant sectors to cocreate health-enabling environments at different levels and tackle complex social determinants of health, such as life-wide education and age-friendly employment.

Moreover, Palauan health systems must target not only sick individuals but also reach out to healthy individuals and provide quality and equitable social and health services. Therefore, care providers must understand each individual and provide customized solutions. This can be achieved by implementing person-centered integrated care, supporting self-management for health, and leveraging technological systems to facilitate communication and sharing of health information among health care and community care workers.

The results of this study show the vital role of communities in fostering healthy aging. Community engagement is a collaborative effort aimed at addressing health concerns and promoting overall well-being for positive and sustainable health outcomes (Rifkin, 2014). At the heart of community engagement is the building and maintenance of relationships between communities and other stakeholders, as well as the utilization of existing community assets (World Health Organization, 2017b). In Palauan communities, these assets can include a strong culture of caring, local organizations providing care programs for older individuals, community-based health programs at old age centers, and a wealth of knowledge and experience from past initiatives. Despite these valuable resources, additional support from the government, private sector, and civil society is necessary to improve sustainability and expand care services. As each community has its own unique resources, needs, and health status of older individuals, it is crucial to ensure equitable access to aging programs and services.

The study found cultural changes around aging with implications for health issues among older persons. This further suggests the importance of a cultural context approach to healthy aging. The perceptions of aging and older adults are dynamic and constantly evolving, shaped by factors such as medical advancements, Western culture, media narratives, and changing demographic trends (Mortimer & Moen, 2016). The language used to describe older persons can also have a significant impact, as research suggests that biased language can perpetuate negative stereotypes and influence attitudes, policies, and health outcomes (Sweetland et al., 2017). The term “elder” in Palau carries positive connotations and aligns with the traditional values of respect and courtesy for those who are advanced in age. Hence, considering the cultural context of aging is crucial in shaping effective and inclusive aging policies, programs, and research in Palau.

Because Palau is still a matrilineal society where men and women have traditional complementary roles, a gender perspective is key to issues of research and policy. This perspective is also relevant to equitable healthy aging in Palau by considering the relationship between gender and health and addressing its differential impact on individuals of all genders. Gender has a significant impact on a person’s health throughout their life, affecting their experiences with crises and emergencies, exposure to diseases, and access to essential resources such as health care, water, hygiene, and sanitation (World Health Organization, 2021). Despite the fact that many health conditions affect individuals of all genders, the rates, trends, and specific types of these conditions differ between genders in Palau. For example, data from 2017 and 2018 show that 36% of school-aged girls and 40% of school-aged boys were overweight or obese (United Nations Women, 2022), and cigarette smoking is more prevalent among men than women (Chiang et al., 2015). Additionally, a higher proportion of women in Palau are known to chew betel nuts with and without tobacco compared to men (55.1% vs 47.5%; Kreisel et al., 2020). Health behaviors and literacy levels also vary between genders in Palau, with men being less likely to participate in health practices, activities, and programs and having lower health literacy levels that discourage them from seeking medical help. Cultural norms play a significant role in gender differences in health and well-being, with women and men being affected differently by customs and rituals in Palau’s matrilineal society. To address the health and social needs of both women and men in Palauan society, a gender-sensitive and gender-inclusive approach should be taken. For instance, advocacy campaigns and peer-to-peer support programs could be considered to encourage more men to adopt healthy lifestyles.

## Conclusion

Our PAR study provides insights into the experiences and needs of older adults in Palauan society, which can inform the development of a comprehensive national aging policy. Our findings highlight the need to optimize social and physical environments to improve the health and well-being of older adults and the importance of a cultural context approach to policymaking around aging. This study also highlights the importance of health systems proactively reaching individuals and communities to address lifelong health needs. These findings have implications for other Pacific Island countries facing similar demographic shifts and provide an opportunity to learn from Palau’s experience in creating age-friendly communities.

## Supplementary Material

gnad078_suppl_Supplementary_Material

## Data Availability

The anonymized data that support the findings of this study are available on request from the corresponding author. The data are not publicly available due to privacy or ethical restrictions. This study was not preregistered.
