# Supplementary material for: Toward Healthy Aging in Palau
Source: Gerontologist. 2023 Jun 23;64(2):gnad078. doi: 10.1093/geront/gnad078 (PMC10825834; doi:10.1093/geront/gnad078)
Supplement: gnad078_suppl_Supplementary_Material [file gnad078_suppl_supplementary_material.docx]

**Online Supplementary Material**

**Supplementary Material 1: Interview guide for focus-group discussions with older adults**

- Focus groups usually last for about 1 to 2 hours. The moderator can provide a short break in between depending on the situation. It is a good practice not to stretch the discussion for too long if the participants do not feel comfortable sitting after a certain time.
- The members of the focus group should have attributes that are homogenous, while there should also remain some degree of variation. Common factors where homogeneity is sought are – occupation, educational level, age, gender, education, or family characteristics.
- Typically, the size of the focus group is from 6 to 8 participants. A mini focus group can consist of fewer participants – usually four or five.
- There should be a pre-defined confidentiality statement, informing participants not to repeat what is said in the focus group to others.
- Pay attention to the nature of the sitting arrangement, size of the table, proximity to each other, and lighting condition.

The demographic information needs to be collected individually over the phone prior to the interview to ensure confidentiality.

1. Age
2. Gender
3. Marital status – Are you single? Married? Widowed? Divorced? Prefer not to say?
4. Language – what is/are your main language(s)? (Language will be used for grouping focus-group participants)
5. Household composition – Do you live on your own or with relatives and/or friends nearby?
6. Socio-economic status

- What is your annual household income?
- What is your educational background?

1. Health condition (For the following question, ask if the person prefers not to respond while asking them)
   - Are you living with any chronic illness?
   - How do you describe your health status (excellent, good, fair, or poor)?
2. Cultural identity/religion. (For these two following questions, ask if the person prefers not to respond while asking them).
   - Do you practice any religion? If so, which one?
   - Do you identify with a particular minority, identity, an ethnic or cultural group in Palau? If so, which one(s)?

Interview Questions: This study will include 4 focus groups (Palauan) and 2 focus groups (non-Palauan). Two of 4 focus groups (Palauan) will focus on environmental aspects of aging, while the other two focus groups (Palauan) will focus on care issues for older adults. The two focus groups (non-Palauan) will go through all the following questions.

**Topic 1: Image of aging**

- Have you experienced any age-related declines?
- Do you feel that people treat you differently now than they did when you were 40 years old?

**Topic 2: Social and physical environments of older adults**

- What is your experience of late-working life, civil engagement, and social inclusion in community activities?
- What is your experience of public transportation, outdoor spaces, and housing? Any unmet needs? existing challenges?
- According to you, how could older adults contribute to the community and society? and what support would you need? Ask to give an example of older adults being active in the community.

**Topic 3: Health and social care services**

- What is your experience of accessing and using health services and treatment? Any unmet needs? existing challenges?
- What is your experience of social services and activities (e.g., group exercise, walking clubs, life-story exercises, lecturers, art classes)? Any unmet needs? existing challenges?
- What could your community/municipality do more to support your health? (Physical, mental and cognitive health)
- What do you do to keep yourself healthy?

**Topic 4: Long-term care**

- What are the activities you typically need help with? Who is helping you?
  - *If it’s a family relative, ask* How do you feel about the care you receive from family relatives (Pros & cons, challenges, unmet needs)
  - *If it’s an in/formal care worker, ask* How do you feel about the care you receive from care workers (Pros & cons, challenges, unmet needs)
- Are you providing care for anyone (e.g., spouse, adult children, grandchildren)? If so, what is your experience of that? (Pros & cons, challenges, and needs)
- Is there anything you want to elaborate on that we have not asked yet?

**Supplementary Material 2: Break-out group instructions**

Groups 1 & 2 - Age-Friendly Environments (Social & Physical)

Health and well-being are determined not only by our genes and personal characteristics but also by the physical and social environments in which we live our lives. Environments play an important role in determining our physical and mental capacity across a person’s life course and into older age and also how well we adjust to the loss of function and other forms of adversity that we may experience at different stages of life, and in particular in later years. Both older adults and the environments in which they live are diverse, dynamic, and changing. In interaction with each other, they (social & physical environments) hold incredible potential for enabling or constraining healthy aging.

Answer the following key questions:

Social environment (labor market, education, civic participation, etc.)

1. Given your community’s context, what might be some ways/ideas to help improve the social environment for older adults?
2. What entities/organizations/agencies would be best suited to deliver & implement which types of social programs for older adults?

Physical environment (housing, transportation, outdoor spaces, etc.)

1. What features are needed to make housing in Palau more age-friendly?
2. What adaptations are needed to accommodate the future needs of your own family in your home? (Address mobility, security, safety, aesthetics – e.g., green/blue spaces, etc.).
3. What are some ideas for how transportation can be improved to support older adults’ needs?
4. What are your views and reflections on this concept of “lifelong learning,” and what might be a way to facilitate new skills development for older adults?
5. Is the digital divide* an issue for older adults in Palau? If yes, what are the root causes of the digital divide? Is it primarily a lack of skills? physical limitations? financial? perception issues (e.g., self-directed ageism)? Or other?
6. What are some ideas for how to bridge the divide to enable access to online public services, civic engagement, and social participation opportunities for older adults? **Digital divide - The term refers to the tendency for older adults to be less likely to use the internet than younger people, and for those older adults who do access the internet to do so less.*

Groups 3 & 4 – Combatting Ageism (Cultural Aspects of Aging)

Ageism affects how we think, feel, and act towards others and ourselves based on age. It imposes powerful barriers to the development of good policies and programs for older and younger people and has profound negative consequences on older adults’ health and well-being.

Answer the following key questions:

1. What are some of the negative cultural mindsets or trends you see in Palau towards older adults that should be addressed urgently?
2. What are some ideas for addressing these problematic cultural mindsets or trends?
3. Are there any positive cultural mindsets or trends you see that we should preserve, maintain or accelerate?
4. What do you think are some of the root causes of cultural changes in how older adults are treated and valued?
5. What are some things we can do to maintain Palauan cultural values of love, respect, and a sense of family responsibility in caring for older adults?
6. How can we design assistance services and programs for older adults that will ensure a healthy balance of support from family, community, and government?

**Supplementary Material 3: Instructions for vision exercise**

Vision Statements are usually aspirational and inspirational, written in the present tense, and describe the desired future state of being. Here are some examples below:

Sample Vision Statements:

- Japan has the vision of creating harmonious communities with enhanced intergenerational solidarity and integrated care.
- Korea has a vision of all age groups living happily in a sustainable society.
- Singapore - a Nation for All Ages – an inclusive home for all Singaporeans and an icon for successful aging in Asia.
- World Health Organization Regional Action Plan for the Western Pacific - healthier older adults in the Western Pacific Region are thriving and contributing to society (“Turning silver into gold”).

Through a thematic analysis of focus-group interviews with older adults, six distinct areas were identified. The analytical process followed a four-step approach, which involved gaining a basic understanding of the experiences and needs of older adults, synthesizing the data to uncover patterns, utilizing the WHO Regional Action Plan on Healthy Aging to contextualize the identified patterns, and ultimately organizing the findings into six thematic areas related to social and health systems.

1. Cultural changes around aging and older adults
2. Social and physical environments
3. Preventive health and health promotion
4. Long-term care
5. Social services for older adults
6. Healthcare

Key questions to answer:

1. If Palau implemented everything it needs to over the next 25 years to achieve healthy aging and age-friendly environments, describe what the year 2050 looks like. (Think about the six action areas below)
2. Based on our six thematic action areas, what is the sub-vision/objective they want for each area?
3. Propose/identify/suggest example interventions and initiatives that help to achieve the sub-vision/objective.

**Supplementary Material 4. Guide for stakeholder interviews**

This guide aims to support capturing perspectives from a diverse group of stakeholders, such as the council on aging, health and care administrators, and political leaders. With the support of the Ministry of Health and Human Services, the following stakeholders are identified as potential study participants.

- One-on-one interviews usually last for about 30 to 60 minutes.
- 8-12 interviews might be sufficient, depending on the status of data saturation.
- Questions are subject to modifications/changes, given the backgrounds of interviewees.
- Produce the best possible audio recordings if conditions allow or well-written notes.

The information needs to be collected prior to interviews: organizational affiliation, years of experience with issues of aging (or older adults), and current work or the field of engagement.

Interview questions:

1. What healthy aging activities do you have now? and what do you have in plans?

(Prompts: care services, income-generating / voluntary opportunities, healthy aging campaigns, professional development of healthcare workforce)

1. How successful have been the programs? What were the challenges?
2. In your view, in which areas would you put more effort? (Prompts: resource scarcity, the effectiveness of policies and programs, prioritizing and targeting issues, implementation problems, universal health coverage, evidence building, knowledge sharing, etc.)
3. (Questions for care providers): What do you think about the status of long-term care for those who need it? (Prompts: older adults’ self-help, eldercare services purchased, family care, tax-funded welfare policy)
4. (Questions for policymakers): What is the current status of multi-sectoral/organizational collaboration? What challenges are there? and what support do you need to enable future multisectoral collaboration?
5. What are the future prospects of your organization in promoting healthy aging? (Prompts: challenges and opportunities in e.g., financial and human resources, individual planning and public engagement, innovations)

**Supplementary Material 5. Preliminary results on older adults' circumstances and needs (approved by Government)**

| **Thematic findings 1: Cultural changes around aging and older persons** | **Thematic findings 2: Social and physical environments** | **Thematic findings 3: Health promotion and disease prevention** |
| --- | --- | --- |
| Positive cultural perceptions about older people are dominant but declining.   - Respect for and attentive to older people - Compassionate ageism and self-directed ageism (which is unique to Palau)   Some negative stereotypes of aging / older people exist.   - Incapable of doing activities/exercises - Inevitably experience dementia, memory disorder or depression   Age/retirement doesn’t define an older person   - Older persons can contribute to family, community and society when they are encouraged and supported to do so. - Older people have diverse personal inspirations/wishes/preferences. | Social participation   - Old age centers are seen as a good opportunity, but not all communities have or utilize them. - Discontinuity of aging programs due to lack of human and financial resources. - Some older people experience social isolation/loneliness.   Income-generating activities   - Negative social perceptions around the employability of older workers - Lack of support system in the labor market for older people   Social inclusion   - Some barriers exist in civil engagement. - Limited access to information about existing services and programs   Transportation and mobility   - Mobility is key to the quality of life and well-being in later life. - Interior design is not friendly to people living with disability/mobility issues. - Under-developed public transportation system   Outdoor spaces   - Few good sidewalks/walkways for older persons and other generations – limited walkability and concerns about safety. - There’s a need for more green beautiful outdoor spaces | - Some preventive services are available, but we need more equitable access. - There is limited coordination, monitoring, and communication for preventive services. - There is a relatively low level of health-promoting behaviors, which prevents addressing risk factors of noncommunicable diseases. - Lack of individualized health planning for community members. |

| **Thematic findings 4: Long-term care needs** | **Thematic findings 5: Social services for older persons** | **Thematic findings 6: Healthcare** |
| --- | --- | --- |
| Young people have work responsibilities / are moving or working away from their parents.  Rising concerns   - Not able to provide care for older people - Some older people see themselves as a family burden - Need for high-quality family care - Caregivers’ health and well-being are worrisome (need for respite care)   Expectations   - Alternative ways (e.g., domestic helpers/caregivers, nursing homes, assisted living, elder day-care centers) - More training for quality care | Social activities may help deliver better health and well-being.   - Group activity 🡪 reduced social isolation and loneliness in later life - Intergenerational activities 🡪 positive health outcomes for all   Social activities may enhance social care for older people and advance development & solidarity.  The community may plan, lead and implement social activities/programs/services. | - Basic and appropriate equipment, medicine and technology are available 🡪 to improve the prevention, diagnosis and treatment of older patients. - Need a geriatric workforce to focus on older adults with multimorbidity, geriatric syndromes, declining physical and cognitive functioning and poor mental health. - Need for a nationally coordinated aging program to enhance integrated care (incl. healthcare). |
